# State Preemption and Local Tobacco Control: Constraints and Opportunities for Innovation in the US

**DOI:** 10.3390/ijerph22060827

**Published:** 2025-05-23

**Authors:** Rishika Chakraborty, Micah L. Berman, Y. Tony Yang, Yan Li, Yan Wang, Debra Bernat, Sabrina Zhang, Carla J. Berg

**Affiliations:** 1 Center for Health Policy and Media Engagement, School of Nursing, George Washington University, Washington, DC 20052, USA; rishikac@gwu.edu (R.C.);; 2 College of Public Health & Moritz College of Law, The Ohio State University, Columbus, OH 43210, USA; berman.31@osu.edu; 3 George Washington Cancer Center, George Washington University, Washington, DC 20052, USA; yanwang20@gwu.edu (Y.W.); dbernat@gwu.edu (D.B.); 4 Department of Epidemiology and Biostatistics, School of Public Health, University of Maryland, College Park, MD 20742, USA; yli6@umd.edu; 5 Joint Program for Survey Methodology, School of Behavioral and Social Science, University of Maryland, College Park, MD 20742, USA; sabrina3@umd.edu; 6 Department of Prevention and Community Health, Milken Institute School of Public Health, George Washington University, Washington, DC 20052, USA; 7 Department of Epidemiology, Milken Institute School of Public Health, George Washington University, Washington, DC 20052, USA

**Keywords:** tobacco control, tobacco policy, health policy, tobacco use, preemption

## Abstract

State preemption of local laws may impede tobacco control, yet little research has examined local policy activity before, during, and after preemption. This study addresses this gap. We summarized state laws preempting local smoke-free workplace, youth access, and licensure laws (CDC’s STATE) and local legislative activity before, during, and after preemption (Americans for Nonsmokers’ Rights Foundation) across 1999–2021. Preemption existed for smoke-free workplaces in 18 states, youth access in 21, and licensure in 13. Regarding smoke-free workplace laws, local laws were passed in 5 of 11 states with preemption throughout; among seven states with partial-period preemption, local laws were enacted before preemption or after repeal in three states but during preemption in two. Regarding youth access, localities adopted laws (e.g., addressing purchase/use/possession or e-cigarettes) in 11 of 18 states with preemption throughout; among the three states with partial-period preemption, localities passed laws before preemption in one state and during preemption in two. For licensure, localities passed laws (e.g., licensing requirements/penalties) in eight of nine states with preemption throughout and three of four states with partial-period preemption. Although state preemption reduced local activity, some localities advanced tobacco control during preemption, underscoring the need for localities to exercise autonomy permitted under preemption.

## 1. Introduction

Some states preempt—or prevent—local communities from passing more stringent or different local laws than at the state level [1,2,3]. Preemption, which can be explicit or implied, has been applied to various policy efforts (e.g., firearm safety and nutrition policies) [1,2,3]. Preemption is not inherently beneficial or harmful. For example, it may be used to ensure that all jurisdictions have a certain threshold of protections [4,5]. However, preemption could also have negative impacts, as local jurisdictions may be better able to address the needs of its community [1,2,3] and often introduce public health policy innovations that may later influence state and national policy [6].

Tobacco control is among the most prominent, long-standing public health applications of state preemption [7]. Evidence-based tobacco control laws that effectively reduce tobacco use and related impacts [8] include the following: (1) prohibiting/restricting tobacco use in public places (e.g., worksites, restaurants, and bars); (2) reducing youth access by prohibiting tobacco sales, access, or distribution to young people; and (3) restricting tobacco retail licensure [6,7]. Notably, tobacco control policies have often been adopted at the state level after being adopted in a critical mass of communities within a state [6]. Communities have adopted and enacted some of the strongest, most innovative, effective tobacco control policies, which have catalyzed shifts in social norms regarding tobacco use [6]. Some reasons for local communities being quicker than states to respond to tobacco-related public health concerns may be that the balance of power between constituent pressure and special interest lobbying and campaign contributions leans toward pro-health legislation [7].

Recognizing the impacts of evidence-based tobacco control policies and the strength of local public health interests [9], in 1985, the tobacco industry initiated efforts to encourage states to preempt (take away the right to pass certain) local tobacco control laws, which often entailed overruling existing laws [1,10,11,12]. The industry has argued that preemption is necessary to preserve business proprietors’ rights and prevent local smoke-free laws from adversely impacting restaurant/bar businesses. After passing the first state tobacco preemption law in 1985, the number of state laws preempting stronger local legislation increased sharply in the 1990s [13].

Tobacco control advocates and practitioners generally agree that preemption adversely impacts tobacco control efforts [14]. Prior research has documented the adverse impacts of the state preemption of smoke-free air policies [15], Tobacco 21 (T21) laws [16], e-cigarette-related laws [17], and other outcomes and related disparities [18]. Because of these impacts, a Healthy People 2030 Tobacco-Use Objective calls to eliminate state laws that preempt stronger local tobacco control laws [19].

However, other research suggests that certain preemption laws could reduce disparities. For example, one study of state T21 law coverage before the passage of the national T21 policy documented that counties with greater percentages of Black individuals and higher poverty rates had lower coverage, whereas counties with higher percentages of Hispanics and college-educated individuals had higher coverage—suggesting that the national T21 law could address these disparities [4]. Additionally, a California-based microsimulation study assessing minimum floor price laws, which may disproportionately impact lower-income consumers, suggested that such laws have the potential to reduce socioeconomic disparities in cigarette smoking prevalence and consumption [5].

This literature underscores the need for research to describe the impacts of preemption, for example, the nature of policy changes at the local level when preemption is in place, before it is implemented, and/or when it is repealed. This study advances this area of research by exploring how localities have navigated and innovated within the constraints of preemption and what has occurred after the repeal of preemption laws, specifically from 1999 to 2021—a period when a large proportion of tobacco preemption laws were enacted and repealed.

## 2. Materials and Methods

This descriptive study analyzed data from 2 sources: (1) the CDC State Tobacco Activities Tracking and Evaluation (STATE) System for tobacco preemption policies and (2) the Americans for Nonsmokers’ Rights Foundation (ANRF) database regarding local tobacco control policies. Two legally trained study team members verified and reviewed each law from the 2 sources using the Westlaw legal database and compiled relevant sections, parts, and/or subparts to characterize and clarify the nature of the laws.

### 2.1. State Tobacco Control Preemption Laws—CDC’s STATE System

We collected data about tobacco preemption policies from CDC’s STATE System [20,21] for each state from 1999 to 2021. The STATE System provides up-to-date tracking of states with laws preempting smoke-free indoor air, youth access to tobacco products, and tobacco licensure regulation. The STATE System’s data are based on CDC quarterly searches of each state’s laws using an online legal research database to identify newly enacted tobacco control legislation. It analyzes and codes the full text of new or amended laws, using CDC-developed criteria (e.g., specific topics of interest, enforcement mechanisms, effective dates, express preemption, etc.). Following analysis and coding of the provisions, the data are subject to a secondary review for quality control. After identified discrepancies are reconciled, the data are embedded within the STATE System and made available online. In determining whether state laws preempt local smoking restrictions, STATE System researchers account for statutes and examine relevant case law, since court rulings at times determine whether local policies are preempted under existing state law.

We identified all states with any period of preemption of smoke-free air, youth access, and licensure laws during the period of 1999–2021. We further categorized the laws as preempting local laws related to the following: (1) smoke-free indoor workplaces; (2) smoke-free restaurants and bars (separately coded but combined for the current paper); (3) youth access (e.g., distribution and sales); and (4) licensure (over-the-counter and vending machines). State tobacco preemption laws use various terms to refer to preemption (e.g., supersede, preempt, uniform, exclusive, or consistent) and may allow grandfathering exceptions or exceptions for particular local jurisdictions [2]. Thus, our team identified the explicit preemption law terminology to understand its application and noted any exceptions (e.g., grandfathering of existing local policies or certain large municipalities not subjected). Next, we coded each year as before preemption, during preemption, or after repeal.

### 2.2. Local Tobacco Control Policies—ANRF’s Database

Local policy data come from ANRF, which maintains a complete repository of tobacco-related ordinances and regulations across the US by date implemented (i.e., effective date) [22]. After identifying all states with any period of preemption of smoke-free air, youth access, and licensure laws during the period of 1999–2021 (using the STATE System), we examined 4 variables across all local jurisdictions within those states to identify localities with local laws pertaining to the following: (1) smoke-free workplaces; (2) smoke-free restaurants and bars; (3) youth access; and (4) licensure. Then, we reviewed each law to assess its nature and application. Finally, we coded the year of enactment (unless they existed before 1999) and summed the number of counties in which newly local laws were adopted each year.

### 2.3. Data Analysis

First, we conducted a descriptive analysis to summarize policies that existed or were implemented at the local level among states with preemption for the entire period or for a proportion of the period by policy domain (i.e., smoke-free indoor air, restaurants/bars, and others; youth access; and licensure). Then, given that the largest number of state preemption laws pertained to smoke-free indoor workplaces and that analyses of the other preemption laws were not feasible due to small sample sizes, we focused on assessing the impact of state preemption of smoke-free indoor workplace policies on local policy changes. We conducted multilevel linear mixed models, accounting for repeated measures within states over time, to evaluate the association between state preemption laws and the presence of local policies over time for the following: (1) workplaces and (2) restaurants/bars (to assess a spillover effect). Random intercepts for states were included to adjust for within-state correlation, allowing each state to have its own baseline level of local policy adoption. Analyses were conducted using SPSS v27.

## 3. Results

Table 1 summarizes key factors for each of the three tobacco control domains (smoke-free air, youth access, and licensure) among states with any preemption laws in 1999–2021. Across the 33 states (66% of all 50 US states) with any preemption laws related to smoke-free air, youth access, or licensure during 1999–2021, 18 states (36% of US states) had preemption laws in one of these domains (Connecticut, Delaware, Florida, Hawaii, Idaho, Illinois, Indiana, Kentucky, Michigan, Mississippi, New Hampshire, New Jersey, Oregon, Rhode Island, South Carolina, Texas, Virginia, and Wyoming), 12 states (24%) had preemption laws in two of these domains (Arizona, California, Delaware, Louisiana, Michigan, Mississippi, Nevada, New Mexico, North Carolina, South Dakota, Utah, and Wisconsin), and 6 states (12%) had preemption laws in three of these domains (Iowa, Oklahoma, Montana, Pennsylvania, Tennessee, and Washington).

### 3.1. Smoke-Free Indoor Workplace Laws

In 1999–2021, 18 states preempted smoke-free air laws pertaining to indoor workplaces during some period. Of the 18 states, 11 had preemption laws that covered the entire period (California, Connecticut, Florida, North Carolina, Oklahoma, Pennsylvania, South Dakota, Tennessee, Utah, Virginia, and Washington), while 7 states covered a proportion of the period (Iowa: 1999–2008; Illinois: 1999–2005; Louisiana: 1999–2006; Montana: 2005–2009; New Jersey: 1999–2006; Nevada: 1999–2006; and Rhode Island: 2005–2006).

#### 3.1.1. States with Smoke-Free Indoor Workplace Preemption Throughout 1999–2021

Among the 11 states with preemption throughout 1999–2021, there were no local smoke-free laws (of any kind) during this time period in 4 states (Florida, Oklahoma, South Dakota, and Tennessee, despite Tennessee’s partial repeal in 2015 for large cities/counties), and Pennsylvania had 1 locality with a smoke-free restaurant law throughout. However, during 1999–2021, smoke-free workplace laws were passed by 9 localities in California, 1 locality each in Connecticut, North Carolina, and Utah, and 13 localities in Washington.

Furthermore, local smoke-free laws for restaurants and/or bars were passed by 17 localities in California, 7 localities in North Carolina, and 1 locality each in Utah and Virginia. In Washington, the smoke-free laws in the 13 localities that covered workplaces also covered restaurants and bars.

#### 3.1.2. States with Smoke-Free Indoor Workplace Preemption During a Proportion of 1999–2021

Among the seven states that preempted smoke-free workplace laws only a proportion of the period, two states had no local smoke-free laws (Nevada and Rhode Island). In 2 states (Iowa and Louisiana), local laws were passed only after repeal (2 localities in Iowa and 21 localities in Louisiana). In Montana, local laws were passed only before preemption (two localities) and after repeal (two localities). Furthermore, smoke-free laws for restaurants/bars were passed in these three states before preemption or after repeal.

In 2 states (Illinois and New Jersey), local smoke-free workplace laws were passed both during preemption and after repeal (2 during preemption and 27 after repeal in Illinois and 3 during preemption and 1 after repeal in New Jersey). Notably, these same 4 localities in New Jersey passed smoke-free restaurant/bar laws in later years, and 30 localities in Illinois passed smoke-free restaurant/bar laws, beginning when preemption was repealed.

Notably, several of the localities that passed smoke-free restaurant/bar laws during preemption periods also included language addressing workplaces. Additionally, other local smoke-free workplace laws passed during preemption included other work settings. However, among the states where preemption was repealed, the year of repeal marked the beginning of increased local legislation.

#### 3.1.3. Regression Results

Table 2 shows the results from the regression analyses assessing the effect of state preemption of smoke-free workplace laws on: (1) local smoke-free workplace laws and (2) local smoke-free restaurant/bar laws. State preemption of smoke-free workplace laws were significantly associated with reduced local policy activity related to smoke-free workplace laws (number of localities with activity: B = −0.372, SE = 0.115, and *p* = 0.002; % of localities: B = −0.003, SE = 0.002, and *p* = 0.064) and restaurant/bar laws (number of localities: B = −0.356, SE = 0.132, and *p* = 0.008; % of localities: B = −0.004, SE = 0.002, and *p* = 0.029) over time.

### 3.2. Other Smoke-Free Air Laws

Three states preempted smoke-free restaurant/bar laws (Delaware: 1999–2002; Michigan: 1999–2021; and New Hampshire: 2003–2021). Oregon passed a law preempting localities from imposing smoke-free restrictions in tobacco specialty stores, cigar bars, etc. (2002–2008; see Table 1 footnote). One state preempted local laws pertaining to government office buildings (Mississippi: 2000–2007).

#### 3.2.1. States with Smoke-Free Preemption Throughout 1999–2021

In Michigan, during preemption, 2 localities passed smoke-free restaurant/bar laws, while 20 passed such laws for workplaces.

#### 3.2.2. States with Smoke-Free Preemption During a Proportion of 1999–2021

In New Hampshire, there were no local smoke-free air laws. In Delaware and Oregon, local laws were passed only outside of preemption periods (one smoke-free workplace law after repeal in Delaware and seven before preemption and two after repeal in Oregon). Three of these jurisdictions in Oregon also implemented smoke-free restaurant/bar laws.

In Mississippi, 13 localities passed smoke-free workplace laws during preemption, and 55 did so after repeal. Additionally, 13 localities in Mississippi passed smoke-free restaurant/bar laws prior to repeal, and 60 passed restaurant/bar laws after repeal.

### 3.3. Youth Access Laws

In 1999–2021, 21 states had some period of preemption on youth access-related laws (Table 3). Of the 21 states, 18 of them had preemption laws that covered the entire period (California, Delaware, Iowa, Indiana, Kentucky, Louisiana, Mississippi, Montana, North Carolina, Nevada, Oklahoma, South Carolina, South Dakota, Tennessee, Utah, Washington, Wisconsin, and Wyoming), while 3 states covered only a proportion of the period (Arizona: 2019–2021; New Mexico: 1999–2020; and Pennsylvania: 2002–2021).

There were no local youth access laws in seven of these states (Delaware, Indiana, North Carolina, Nevada, South Carolina, South Dakota, and Tennessee). Localities had youth access laws throughout the study period (likely aligning with state law) in 11 states. There were no local youth access laws in Louisiana during 1999–2021, except for one local law that existed throughout this period.

#### 3.3.1. States with Youth Access Preemption Laws Throughout 1999–2021

Localities adopted youth access-related laws consistent/redundant with state laws, to address the purchase, use, and/or possession, or to address e-cigarettes in 11 states (California, Iowa, Kentucky, Mississippi, Montana, New Mexico, Oklahoma, Utah, Washington, Wisconsin, and Wyoming).

#### 3.3.2. States with Youth Access Preemption Laws During a Proportion of 1999–2021

Youth access laws were adopted in 2 localities prior to preemption in Arizona, 2 localities during preemption in New Mexico, and 12 localities during preemption in Pennsylvania.

### 3.4. Licensure

In 1999–2021, 13 states had some period of preemption on licensure-related laws. Of the 13 states, 9 of them had preemption laws that covered the entire period (Iowa, Idaho, Michigan, Montana, Oklahoma, Tennessee, Texas, Washington, and Wisconsin), while 4 states covered only a proportion of the period (Arizona: 2019–2021; Hawaii: 2018–2021; New Mexico: 2021–ongoing; and Pennsylvania: 2016–2021).

During this period (1999–2021), there were no local licensure laws in one of these states—Tennessee. Localities in 11 states had local licensure laws in place throughout.

#### 3.4.1. States with Licensure Preemption Laws Throughout 1999–2021

Localities passed local laws during the preemption period, largely pertaining to vending machines, e-cigarettes, additional licensing requirements, or penalties (including for sales to minors), in eight states (Iowa, Idaho, Michigan, Montana, Oklahoma, Texas, Washington, and Wisconsin).

#### 3.4.2. States with Licensure Preemption Laws During a Proportion of 1999–2021

Licensure-related laws were passed prior to preemption in two localities in Arizona, two localities in New Mexico, and one locality in Hawaii. These laws mainly restricted sales of tobacco, including e-cigarettes, to persons under 21.

## 4. Discussion

This study documented local tobacco-related legislative activity in states with laws preempting local smoke-free indoor policies, youth access, and licensure in 1999–2021. During this period, two-thirds of US states had some type of tobacco-related preemption law: 18 states implemented preemption laws for smoke-free workplaces, 5 states had such laws for smoke-free restaurants/bars, 21 states preempted local youth access laws, and 13 states preempted local licensure-related laws. Furthermore, over one-third of US states had preemption laws pertaining to at least two domains (i.e., smoke-free air, youth access, or licensure). Thus, over the past three decades, preemption has been an important tobacco control issue in the US, as it has impeded local efforts which have historically catalyzed tobacco control through innovation and developing evidence for new strategies [16]. Furthermore, preemption may contribute to or exacerbate tobacco-related disparities [3,7,18], and once implemented, these laws are difficult to repeal [16].

Notably, we found that state smoke-free workplace preemption predicted less local policy activity related to smoke-free workplace and restaurant/bar laws. However, we also documented that, following repeal, many states showed increased local smoke-free legislative activity. Moreover, roughly half of local jurisdictions in states with preemption were still active during preemption. Such activity at the local level tended to either bring the following: (1) adopt local laws that were consistent or redundant with the state law or (2) complement or supplement state laws. For example, when a state preempted smoke-free workplace laws, a city might pass a law banning smoking in outdoor parks or near public transit stops. Also, during periods when states preempted local smoke-free workplace laws, localities passed laws that addressed other workplace settings or public places, and when states preempted local youth access or licensure laws, localities passed laws that augmented state laws by including additional penalties related to sales to minors or restrictions related to e-cigarettes. Other research has also documented the important role of local lawmaking efforts when states implement such statewide policies and how local efforts can address disparities in tobacco control policy coverage [23]. For example, a study of California’s 1994 Smoke-free Workplace Act, which had numerous exemptions, or loopholes, that potentially contributed to inequities in smoke-free air protections [23], found that, in 1994–2018, of 16 identified loopholes, local jurisdictions closed an average of six loopholes, by passing laws to restrict tobacco use in outdoor recreational spaces, outdoor hospitality settings, outdoor events (e.g., music festivals), service areas (e.g., taxi stands), and community streets or districts. Local action was particularly prominent in localities that were more populous, had a greater percentage of White residents, higher income, and were more urban, and disparities did not reduce until 2015–2016 [23]. These findings indicate that, despite state preemption, local jurisdictions may find opportunities to advance tobacco control efforts in their communities, especially given that previous research has indicated that local lawmakers and stakeholders perceive preemption as a formidable barrier and often abandon or delay policymaking efforts when faced with preemption [14].

Current findings have limitations that suggest the need for future research. First, this study focused on states with preemption laws in 1999–2021, which captures the period of the most state adoption of preemption laws, but not all such laws or states. Thus, research assessing local legislative activity in states that did not experience any tobacco-related preemption during this period is also warranted, as this may shed light on other factors that may impede local tobacco control efforts. This is particularly critical given that we found no relevant local laws in several of these states, including states that were not covered by preemption throughout this period. Additionally, we were unable to statistically test the impact of state preemption related to youth access and licensure, and we did not fully describe the nature of the state preemption laws or all local laws passed during, before, or after preemption, given the diversity of these policies and the number of localities across these states. This information is important for fully comprehending the impacts of preemption and opportunities for local activity during preemption.

## 5. Conclusions

Results from this study have implications for practice and policy. While this study focused on smoke-free indoor policies, youth access, and licensure broadly, preemption has recently focused on these dimensions applied to e-cigarettes or other newer tobacco products, as well as other policy areas such as firearms and sugar-sweetened beverages. For example, in the US in 2022, 25 states had laws that preempted stricter local e-cigarette regulations (often not explicitly using the term “preempt”) [17], and since 2017, 4 states passed laws preempting local sugar-sweetened beverage taxes, largely instigated by the beverage industry (via front groups, trade associations, and lobbying) [24]. Thus, understanding the prevalence and impacts of preemption laws could inform public health efforts broadly. Ultimately, this study underscores the need to apply frameworks to limit preemption legislation [25,26], for example, by encouraging stakeholders (e.g., citizens and decision-makers) to monitor and anticipate preemption, engage allies and advocacy groups, and revisit policy language to maintain local authority [25,26]. Preserving local authority may enable communities to tailor public health strategies that reflect their specific needs and promote innovation.

## Figures and Tables

**Table 1:** Description of local tobacco control laws among states with state preemption on local smoke-free air laws.

States with Preemption:		No Local Laws or Local Laws Throughout	Local Laws Passed During Preemption in States with Preemption Throughout 1999–2021	Contrasting Distinct Preemption Periods
**Throughout** **1999–2021**	During a Proportion of 1999–2021			
**Smoke-free indoor workplaces, 18 states**				
**11 states**: * California, Connecticut, Florida, North Carolina, Oklahoma, Pennsylvania, South Dakota, Tennessee, Utah, Virginia, Washington	**7 states**: ^ Iowa: 1999–2008; Illinois: 1999–2005; Louisiana: 1999–2006; Montana: 2005–2009; New Jersey: 1999–2006; Nevada: 1999–2006; Rhode Island: 2005–2006	**No local laws—6 states**: ^#^ 4 of the 11 with preemption throughout (Florida, Oklahoma, South Dakota, and Tennessee); 2 of the 7 with proportion of period (Nevada and Rhode Island)	**Local smoke-free workplace laws—5 states:** - California: 9 (2000, 2002, 2007 [2], 2010, 2017, 2018, 2019 [2]) - Connecticut: 1 (2009) - North Carolina: 1 (in 2006) - Utah: 1 (2014) - Washington: 13 (2005, 2006 [3], 2007, 2009, 2011 [2], 2014, 2016 [2], 2019, 2020) **Local smoke-free restaurants/bars—5 states:** - California: 17 (2000, 2001, 2002, 2006 [3], 2007, 2009 [2], 2010, 2012, 2017 [2], 2018, 2019 [3]) - North Carolina: 7 (2006, 2009, 2012 [2], 2016, 2019, 2020) - Utah: 1 (2014) - Virginia: 1 (2007) - Washington: 13 local workplace laws also covered restaurants/bars (same years)	**Local smoke-free workplace laws passed only prior to preemption or after repeal—2 states:** - Iowa: 2 after repeal (2008, 2016) - Montana: 2 prior to preemption (2002); 2 after repeal (2017, 2018) **Local smoke-free workplace laws passed during preemption—3 states:** - Illinois: 2 prior to preemption (2003, 2005); 27 after repeal (2006 [6], 2007 [2], 2008 [10], 2009 [2], 2010 [3], 2103 [2], 2014, 2015) - Louisiana: 6 during preemption (2005 [5], 2006); 15 after repeal (2007 [2], 2011, 2014, 2015 [2], 2016, 2017 [2], 2018, 2019 [5]) - New Jersey: 3 during preemption (2001, 2004, 2005); 1 after repeal (2007) **Local smoke-free restaurants/bars—5 states:** - Iowa: 2 after repeal (2008, 2016) - Illinois: 30 after repeal (2005; 2006 [6]; 2007 [3]; 2008 [10]; 2009 [2]; 2010 [4]; 2013 [2]; 2014; 2015) - Louisiana: 19 after repeal (2008, 2011, 2013, 2014, 2015 [2], 2016, 2017 [3], 2018, 2019 [6], 2020 [2]) - Montana: 2 during preemption (2002 [2]); 2 after repeal (2017, 2018) - New Jersey: 4 that passed workplace laws passed restaurant/bar laws after repeal (2006, 2007, 2008, 2017)
**Preemption on other smoke-free air laws, 5 states**				
**Food service establishments**: Michigan ^§^	**Food service establishments**: Delaware: 1999–2002; New Hampshire: 2003–2021 **Prohibited smoke-free laws on tobacco specialty stores, cigar bars, etc.:** Oregon ^£^: 2002–2008 **Government office buildings**: Mississippi: 2000–2007	New Hampshire	**Local smoke-free workplace or restaurant/bar laws—1 state:** - Michigan: 20 local smoke-free workplace laws (2002, 2005 [4], 2006 [2], 2007 [10], 2009 [2], 2019) - Michigan: 2 local restaurant/bar laws (2019, 2021)	**Local smoke-free workplace laws—3 states:** - Mississippi: 13 during preemption (2002, 2003 [2], 2005, 2006 [4], 2007 [5]); 55 after repeal (2008 [4], 2010 [7], 2011 [9], 2012 [9], 2013 [4], 2014 [5], 2015 [9], 2017 [5], 2019 [2], 2020) - Delaware: 1 after repeal (2016) - Oregon: 7 prior to preemption (2000 [5], 2001 [2]); 2 after repeal (2009, 2018) **Local smoke-free restaurant/bar laws—2 states:** - Mississippi: 13 during preemption (2002, 2005, 2006 [5], 2007 [6]); 60 after repeal (2008 [7], 2009, 2010 [10], 2011 [9], 2012 [9], 2013 [4], 2014 [5], 2015 [8], 2017 [4], 2019 [2], 2020) - Oregon: 1 prior to preemption (2000); 2 after repeal (2009, 2016)

Notes: * Florida (ceiling for smoking but not vaping); Oklahoma (in 2013, amended to exclude government property); Pennsylvania (does not apply to 1st-class cities); Tennessee (in 2015, partial repeal for large cities or counties). ^ Louisiana (originally office workplaces; in 2003, added restaurants, bars, and gaming). ^#^ 1 state had 1 local law (for restaurants) throughout: Pennsylvania. ^§^ Explicitly included bars. ^£^ Tobacco specialty stores and bars/taverns/restaurant sections off-limit to minors as well as gambling/establishments used for bingo, bowling centers, designated hotel smoking areas, and specific employee lounges.

**Table 2:** Regression analyses assessing the effect of state preemption of local smoke-free workplace laws on local changes in smoke-free workplace and restaurant/bar laws, separately, *n* = 18 states.

**Number of Counties in Each State that Enacted:**	**Local Smoke-Free Workplace Laws**			**Local Smoke-Free Restaurant/Bar Laws**		
Predictor	**B (SE)**	**CI**	***p***	**B (SE)**	**CI**	***p***
State smoke-free workplace preemption	−0.372 (0.115)	−0.601, −0.144	0.002	−0.356 (0.132)	−0.618, 0.095	0.008
**Percent of Counties in Each State that Enacted:**	**Local Smoke-Free Workplace Laws**			**Local Smoke-Free Restaurant/Bar Laws**		
Predictor	**B (SE)**	**CI**	***p***	**B (SE)**	**CI**	***p***
State smoke-free workplace preemption	−0.003 (0.002)	−0.007, 0.000	0.064	−0.004 (0.002)	−0.008, 0.000	0.029

Notes: Controlling for time and accounting for repeated measures within states.

**Table 3:** Description of local tobacco control laws among states with state preemption on local youth access and licensure laws.

States with Preemption:		No Local Laws or Local Laws Throughout	Local Laws Passed During Preemption in States with Preemption Throughout 1999–2021	Contrasting Distinct Preemption Periods
Throughout 1999–2021	A Proportion of 1999–2021			
**Youth access, 21 states**				
**18 states:** California, Delaware, Iowa, Indiana, Kentucky, Louisiana, Mississippi, Montana, North Carolina, Nevada, Oklahoma, South Carolina, South Dakota, Tennessee, Utah, Washington, Wisconsin, Wyoming	**3 states:** Arizona: 2019–2021; New Mexico: 1999–2020; Pennsylvania: 2002–2021	**No local laws—7 states:** all in states with preemption throughout (Delaware, Indiana, North Carolina, Nevada, South Carolina, South Dakota, and Tennessee) **Local laws throughout—11 states: ^§^** Arizona: 1; California: 3; Iowa: 1; Louisiana: 1; Mississippi: 7; Montana: 1; Oklahoma: 2; Pennsylvania: 1; Washington: 3; Wisconsin: 21; Wyoming: 2	**Local laws consistent with state laws, related to purchase, use, and/or possession, and/or addressing e-cigarettes—11 states:** - California ^: 11 (2003, 2013, 2014 [2], 2015, 2016, 2017, 2018, 2019 [3]) - Iowa: 2 (2000, 2014) - Kentucky: 1 (2014) - Mississippi *: 2 (2016, 2020) - Montana ^: 2 (2020, 2021) - New Mexico: 2 (2000, 2002) - Oklahoma: 5 (2000 [2], 2007, 2009, 2010, 2012, 2013) - Utah: 1 (2014) - Washington: 4 (2011, 2014 [2], 2015) - Wisconsin: 8 (2000 [2], 2003, 2007, 2009, 2012, 2015, 2018) - Wyoming: 3 (2000 [2], 2008)	**Local laws passed prior to preemption—1 state:** - Arizona: 2 (in 2018) **Local laws passed during preemption—2 states:** - New Mexico ^: 2 (2000, 2002) - Pennsylvania ^: 12 (2003 [3], 2004 [2], 2005, 2006, 2007, 2008, 2011, 2014, 2020)
**Licensure, 13 states**				
**9 states:** Iowa, Idaho, Michigan, Montana, Oklahoma, Tennessee, Texas, Washington, Wisconsin	**4 states:** Arizona: 2019–2021; Hawaii: 2018–2021; New Mexico: 2021–ongoing; Pennsylvania: 2016–2021	**No local laws—1 state:** Tennessee **Local laws throughout—11 states:** Arizona: 1; Hawaii: 2; Iowa: 2; Idaho: 1; Michigan: 10; Montana: 1; Oklahoma: 2; Pennsylvania: 8; Texas: 8; Washington: 3; Wisconsin: 20	**Local laws for licensing requirements, penalties, vending machines, e-cigarettes, etc.—8 states:** - Iowa: 3 (2000 [2], 2012) - Idaho ^#^: 1 (2018) - Michigan ^¥^: 6 (2013, 2014, 2017, 2018 [2], 2020) - Montana: 1 (2020) - Oklahoma ^¥^: 8 (2000, 2007, 2008, 2009, 2010, 2012, 2013, 2019) - Texas: 16 (2014 [9], 2015 [2], 2017 [2], 2018 [3]) - Washington: 7 (2006, 2009, 2011 [2], 2014 [2], 2015) - Wisconsin: 9 (2003 [3], 2004, 2005, 2007 [3], 2015)	**Local laws passed prior to preemption—3 states:** - Arizona: 2 (2016, 2018) - New Mexico ^¥^: 2 (2000, 2011, 2014) - Hawaii ^≠^: 1 (2013)

Notes: ^§^ Louisiana had no other local laws. * Also specified minimum age of 21. ^ Localities passed laws regarding youth access (California: 2013; Montana: 2021; New Mexico: 2021 [3]; Pennsylvania: 2020). ^#^ Restricted sales of tobacco, including e-cigarettes, to persons < 21; this was repealed. ^¥^ Localities passed laws regarding cannabis (Michigan; Oklahoma: 2019; New Mexico: 2021 [3]). ^≠^ Restricted sales of tobacco, including e-cigarettes, to persons < 21 (except if 18 by 6/30/14).

## Data Availability

The original contributions presented in this study are included in the article. Further inquiries can be directed to the corresponding author.
